# Reply: Antibody levels against BK virus and prostate, kidney and bladder cancers in the EPIC-Oxford cohort

**DOI:** 10.1038/sj.bjc.6603125

**Published:** 2006-06-13

**Authors:** R Newton, P Coursaget, I S D Roberts

**Affiliations:** 1 Epidemiology & Genetics Unit, Department of Health Sciences, University of York, Area 3, Seebohm Rowntree Building, Heslington, York YO10 5DD, UK; 2 INSERM U618, Faculté de Pharmacie, 37200 Tours, France; 3 Department of Cellular Pathology, John Radcliffe Hospital, Headington, Oxford OX3 9DU, UK

**Sir,**

An investigation of anti-BK virus antibodies among immunosuppressed patients with BK-related nephropathy was beyond the scope of the EPIC-Oxford cohort study (Newton *et al*, 2005), but has been carried out by others. Hariharan *et al* (2005) found that mean BK virus-specific IgG levels in patients with early onset and stabilizing BK virus nephropathy were significantly lower than those seen after the disease had resolved. Mean plasma BK virus load declined over the same period. Chen *et al* (2006) observed that low anti-BK virus antibodies were associated with a low viral load and strong cytotoxic T lymphocyte (CTL) response and that viral persistence among transplant recipients was associated with high antibody titres and a poor CTL response. However, for results to be truly comparable to those reported by us, antibody titres among such patients would need to be assessed using the same assay, preferably performed at the same time and under the same laboratory conditions.

We agree that our prospective investigation does not exclude the possibility of a ‘hit-and-run’ role for BK virus in the aetiology of any of the cancers studied. However, there is no example of such a mechanism in the aetiology of cancer. Indeed among patients with cancers that are known to be related to prevalent infections, such as Kaposi's sarcoma (human herpesvirus 8), cancer of the uterine cervix (human papillomaviruses) or endemic African Burkitt lymphoma (Epstein Barr virus), elevated titres of antibodies against the relevant viral antigens have been found both in prospective and in retrospective studies (for examples see references: de-Thé *et al*, 1978; Sitas *et al*, 1999; Newton *et al*, 2004; Newton *et al*, in press).

However, we can also report some additional evidence against a role for BK virus in the aetiology of prostate cancer. Using immunohistochemistry, 10 consecutive prostate cancer tissue sections were incubated with antibodies to the SV40 T antigen. This antibody cross reacts with the BK virus large T-antigen expressed on infected cells and is used routinely to identify polyoma virus infection among transplant recipients with haemorrhagic cystitis or nephropathy (Mann and Carroll, 1984). Presence of viral protein is associated with characteristic nuclear staining. None of the samples tested were positive for SV40T antigen, suggesting that BK viral proteins are not expressed on tumour cells. These results are in accord with those of a previous study, which also reported negative results for bladder cancer (Shah *et al*, 1978). Lack of BK virus protein does not exclude the possibility that viral DNA is present, although previous studies designed to identify BK virus genomes in normal prostate tissue and in prostate cancer cells have produced inconsistent results and are difficult to interpret (Zambrano *et al*, 2002; Das *et al*, 2004).

In relation to bladder cancer, on the basis of only nine cases, we found no evidence that anti-BK virus antibodies were associated with the tumour. Only one published study has reported finding SV40 T antigen present in tumour tissue (but not in adjacent normal tissue), but this was an unusual case in an immunosuppressed transplant recipient (Geetha *et al*, 2002). Studies of bladder cancer in immunocompetent people have not found SV40 T antigen (Shah *et al*, 1978). BK virus DNA has been identified in papillary urothelial bladder carcinoma in one study (Monini *et al*, 1995).

A common morphological sign of active polyoma virus infection is the presence in urine of bladder epithelial cells with typical intranuclear viral inclusions. These ‘decoy cells’ are so named because they mimic the nuclear enlargement and atypia of urothelial carcinomas and can easily be mistaken for malignant cells. Weinreb draws attention to his recent report (Weinreb *et al*, 2006) in which decoy cells were identified in urine cytology specimens (using the Papinicolaou method) of apparently immunocompetent people who subsequently developed bladder cancer. While interesting, the interpretation of this finding is uncertain. In order to exclude a diagnosis of cancer and confirm BK virus infection, the presence of decoy cells on urine cytology should be followed by staining for SV40 T antigen. No such confirmatory tests were reported by Weinreb and co-workers. Although his study was prospective, no detail is provided about the time between urine cytology and diagnosis of bladder cancer and we suggest that the decoy cells could in fact have been malignant cells, despite the authors' assertion to the contrary. Urine samples were not available in the EPIC-Oxford cohort.
